# MRI and fluid biomarkers reveal determinants of myelin and axonal loss with aging

**DOI:** 10.1002/acn3.51730

**Published:** 2023-02-10

**Authors:** Keenan A. Walker, Michael R. Duggan, Zhaoyuan Gong, Heather E. Dark, John P. Laporte, Mary E. Faulkner, Yang An, Alexandria Lewis, Abhay R. Moghekar, Susan M. Resnick, Mustapha Bouhrara

**Affiliations:** ^1^ Laboratory of Behavioral Neuroscience, National Institute on Aging National Institutes of Health Baltimore Maryland 21224; ^2^ Laboratory of Clinical Investigation, National Institute on Aging National Institutes of Health Baltimore Maryland 21224; ^3^ Department of Neurology Johns Hopkins University School of Medicine Baltimore Maryland 21224

## Abstract

**Objective:**

White matter damage is a feature of Alzheimer's disease, yet little is known about how facets of the Alzheimer's disease process relate to key features of white matter structure. We examined the association of Alzheimer's disease (Aß_42/40_ ratio; pTau181), neuronal injury (NfL), and reactive astrogliosis (GFAP) biomarkers with MRI measures of myelin content and axonal density.

**Methods:**

Among cognitively normal participants in the BLSA and GESTALT studies who received MRI measures of myelin content (defined by myelin water fraction [MWF]) and axonal density (defined by neurite density index [NDI]), we quantified plasma levels of Aβ_42_, Aβ_40_, pTau181, NfL, and GFAP. Linear regression models adjusted for demographic variables were used to relate these plasma biomarker levels to the MRI measures.

**Results:**

In total, 119 participants received MWF imaging (age: 56 [SD 21]), of which 43 received NDI imaging (age: 50 [SD 18]). We found no relationship between plasma biomarkers and total brain myelin content. However, secondary analysis found higher GFAP was associated with lower MWF in the temporal lobes (*ß* = −0.13; *P =* 0.049). Further, higher levels of NfL (*ß* = −0.22; *P =* 0.009) and GFAP (*ß* = −0.29; *P =* 0.002) were associated with lower total brain axonal density. Secondary analyses found lower Aβ_42/40_ ratio and higher pTau181 were also associated with lower axonal density, but only in select brain regions. These results remained similar after additionally adjusting for cardiovascular risk factors.

**Interpretation:**

Plasma biomarkers of neuronal injury and astrogliosis are associated with reduced axonal density and region‐specific myelin content. Axonal loss and demyelination may co‐occur with neurodegeneration and astrogliosis ahead of clinically meaningful cognitive decline.

## Introduction

White matter damage and degradation are pervasive features of Alzheimer's disease (AD) known to occur very early in the disease course.^1^ White matter abnormalities have been associated with increased risk of future AD dementia and cognitive decline,^2^, ^3^ and there is considerable evidence for a causal link between white matter dysfunction and AD.^4^, ^5^ Degradation of myelin sheaths, myelin‐producing oligodendrocytes, and axonal damage are considered primary and proximal causes of white matter damage, including white matter hyperintensities (WMHs) often observed using structural neuroimaging.^6^, ^7^, ^8^

The structural integrity of white matter is commonly measured by quantifying white matter lesions using T2/FLAIR MRI and white matter microstructural alterations using diffusion tensor imaging (DTI). Although parameters derived from these MR imaging techniques have been found to be very sensitive to cerebral microstructural changes with aging and neurodegeneration,^9^, ^10^, ^11^ the underlying structural mechanisms responsible for these findings are difficult to define due to the sensitivity of derived indices to a number of tissue properties, including hydration, macromolecular content, axonal density, myelin content, iron content, and architectural features such as fiber crossing and fanning. To address these limitations, more advanced analytic methods based on multicomponent relaxometry or diffusion have been introduced to improve both sensitivity and specificity of MR‐based myelination and axonal density studies.^12^, ^13^ These approaches have been applied to characterize a variety of cerebral diseases and neurodevelopment.^14^, ^15^, ^16^, ^17^

While multicomponent relaxometry approaches permit specific quantification of the myelin water fraction (MWF), a surrogate of myelin content, they are unable to distinguish between intracellular water fraction, a proxy of axonal density, and extracellular water fraction due to their similar relaxation times. To overcome this difficulty, multicomponent diffusion techniques to map intracellular water in brain have been introduced.^18^ These techniques are based on compartmental models of water diffusion incorporating intracellular water (i.e., water within neurites which is a proxy of axonal density in WM), extracellular water, and a compartment consisting of less restricted water from the cerebrospinal fluid volume. These techniques have been shown to provide reliable and specific information about neurite density in studies of aging,^19^, ^20^, ^21^ as well as in investigations of various neurologic disorders.^22^, ^23^ Therefore, combining multicomponent relaxometry and diffusion provides a unique venue to investigate cerebral changes in aging and neurodegeneration with high sensitivity and specificity. Using our advanced method, BMC‐mcDESPOT,^24^, ^25^, ^26^ to quantify MWF, our group has recently shown that compared to neurologically healthy older adults, those with mild cognitive impairment (MCI), AD dementia, and vascular dementia each had lower myelin content.^14^ A separate study found an association between lower MWF and higher indices of AD pathology in CSF among asymptomatic individuals.^27^

Closely coupled with the health of myelin is axonal integrity. The recent introduction of neurite orientation dispersion and density imaging (NODDI), a multicomponent diffusion technique, provides the opportunity to measure various aspects of neurite microstructure with a high degree of sensitivity and specificity.^18^, ^22^, ^28^, ^29^ The neurite density index (NDI) derived from NODDI provides a microstructural quantification of neurite or axonal density that has been found to decline in the context of AD and relate to atrophy, cortical tau, and cognition.^30^, ^31^, ^32^

Although a relationship between myelin content, axonal density and AD has been demonstrated, less is known about the extent to which these white matter features relate to distinct components of AD pathogenesis, particularly in the preclinical (asymptomatic) phase of the disease process. By examining the relationship between myelin content, axonal density, and biomarkers of AD pathology and non‐specific features of AD, such as neuronal injury and astrogliosis, we can better understand how distinct facets of the AD process relate to white matter degradation. Using data from two National Institute on Aging cohorts, the current study examined the association of plasma biomarkers of AD pathology (Aβ_42/40_, pTau181), neuronal injury (NfL), and reactive astrogliosis (GFAP) with state‐of‐the art MRI measures of myelin content (MWF) and axonal density (NDI) across a wide age range. We hypothesized that greater levels of biomarker‐defined AD pathology, neuronal injury, and astrogliosis would be associated with reduced myelin content or axonal density, particularly among older adults.

## Methods

### Study cohort

Cognitively normal participants were drawn from the Baltimore Longitudinal Study of Aging (BLSA) and the Genetic and Epigenetic Signatures of Translational Aging Laboratory Testing (GESTALT) study.^33^, ^34^ The goals of the BLSA and GESTALT studies include evaluation of multiple biomarkers related to aging, and their inclusion and exclusion criteria are similar. Participants were excluded if they had metallic implants, or major neurologic or medical disorders. Experimental procedures were performed in compliance with our local Institutional Review Board, and participants provided written informed consent.

### MR imaging

MRI scans were performed on a 3T whole body Philips MRI system (Achieva, Best, The Netherlands) using the internal quadrature body coil for transmission and an eight‐channel phased‐array head coil for reception. BLSA and GESTALT participants were scanned using the same MRI system and MRI protocols and sequences. Each participant underwent:

- Our BMC‐mcDESPOT protocol for MWF.^25^, ^26^ This imaging protocol consisted of 3D spoiled gradient recalled echo (SPGR) images acquired with flip angles (FAs) of [2 4 6 8 10 12 14 16 18 20]°, echo time (TE) of 1.37 ms, repetition time (TR) of 5 ms, with an acquisition time of ~5 min, as well as 3D balanced steady state free precession (bSSFP) images acquired with FAs of [2 4 7 11 16 24 32 40 50 60]°, TE of 2.8 ms, TR of 5.8 ms, and acquisition time of ~6 min. The bSSFP images were acquired with radiofrequency (RF) excitation pulse phase increments of 0 or π to account for off‐resonance effects for a total scan time of ~12 min. All SPGR and bSSFP images were acquired with an acquisition matrix of 150 × 130 × 94, voxel size 1.6 × 1.6 × 1.6 mm. Further, we used the double‐angle method (DAM) to correct for the excitation RF inhomogeneity.^35^ We acquired two fast spin‐echo images with FAs of 45° and 90°, TE of 102 ms, TR of 3000 ms, acquisition voxel size of 2.6 × 2.6 × 4 mm, and acquisition time of ~4 min. All images were acquired with a field‐of‐view (FoV) of 240 × 208 × 150 mm, SENSE factor of 2, and reconstructed to a voxel size of 1 × 1 × 1 mm. The total acquisition time was ~21 min.
- Our NODDI protocol for NDI mapping has been published previously.^29^ This imaging protocol consisted of 2D diffusion‐weighted images (DWI) acquired using a single‐shot echo planar imaging sequence: repetition time (TR) / echo time (TE) of 10,000/67 ms, two *b*‐values of 700 and 2000 s/mm^2^, each encoded in 32 diffusion‐weighting gradient directions, FoV of 240 × 208 × 150 mm, acquisition matrix of 80 × 70 × 50, acquisition voxel size of 3 × 3 × 3 mm, and SENSE factor of 2. Two images obtained at *b* = 0 s^2^/mm were acquired and averaged. All images were reconstructed to a voxel size of 2 × 2 × 2 mm. The total acquisition time was ~16 min.

### Myelin water fraction and neurite density imaging mapping

For each participant, an MWF map was generated using the BMC‐mcDESPOT analysis,^24^, ^25^, ^26^ from the SPGR, bSSFP, and DAM images that were linearly registered to the SPGR image acquired at FA of 10^o^. An NDI map was generated using the original NODDI analysis as described previously.^18^ Briefly, NDI was derived from the corresponding DW images that were registered to the *b*_*0*_ image and corrected for motion and eddy‐current‐induced distortion artifacts using the Artifact Correction in Diffusion MRI (ACID) toolbox.^36^

### Regions‐of‐interest segmentation

**Figure 1:**
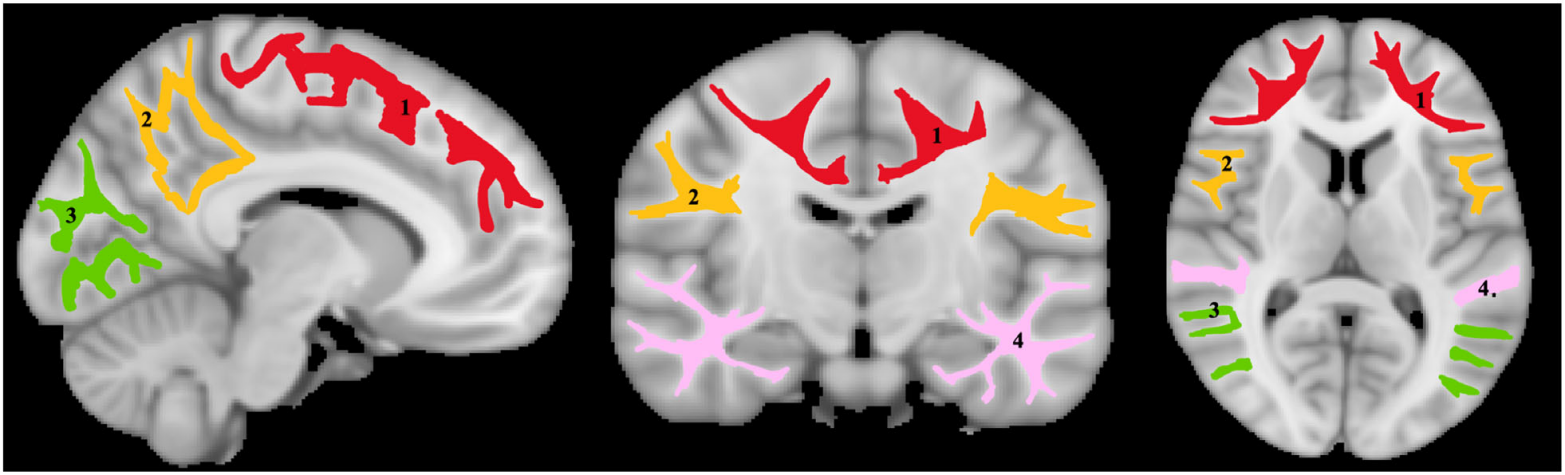
Visualization of the white matter ROIs investigated in our analysis. (1) frontal lobe white matter, (2) parietal lobe white matter, (3) occipital lobe white mater and (4) temporal lobe white matter.

The averaged SPGR image over FAs for each participant was registered using nonlinear registration to the Montreal Neurological Institute (MNI) standard space image and the derived transformation matrix was then applied to the MWF map for that participant. Similarly, the averaged DW image obtained with *b* = 0 was registered using nonlinear registration to the MNI image and the derived transformation matrix was then applied to the NDI map. Five white matter regions of interest (ROIs) were defined from the MNI structural atlas corresponding to the whole brain (WB), frontal lobes (FL), parietal lobes (PL), temporal lobes (TL), and occipital lobes (OL) (see Fig. 1). All ROIs were eroded to reduce partial volume effects and imperfect image registration using the FSL tool *fslmaths*. Finally, the mean MWF and NDI values in each ROI of each participant were calculated.

### Plasma biomarkers

Blood for plasma biomarker measurement was collected at the time of 3 T MRI scan. Plasma was separated, aliquoted and stored at −80°C using standardized protocols. EDTA plasma was used to measure Aβ_42_, Aβ_40_, glial fibrillary acidic protein (GFAP), neurofilament light chain (NfL) using the Quanterix Single Molecule Array (Simoa) Neurology 4‐Plex E assay on the HD‐X Instrument (Quanterix Corporation). Phosphorylated tau‐181 (pTau181) was measured using the Quanterix Simoa p‐Tau181 Assay version 2 on the HD‐X Instrument. All assays were run in duplicate, and the mean value of the duplicate measurements was used for all analyses. Biomarkers derived from BLSA and GESTALT were quantified using the same set of assays (identical lot numbers). The intraassay coefficients of variation (CV) as derived from the parent study were 1.9, 2.8, 5.0, 5.1, and 4.4 for measures of Aβ_42_, Aβ_40_, GFAP, NfL, and pTau181, respectively. One NfL measurement was considered an outlier based on visual inspection and was removed from subsequent analyses.

### Statistical analysis

Multiple linear regression was used to investigate the regional associations between MWF and NDI MR imaging indices (independent variable) and plasma biomarkers (dependent variable). Independent variables included mean MWF or NDI value within each ROI, and covariates included sex, age, and race. Plasma biomarkers values were log‐transformed to remove the skewness of their distributions. Further, to facilitate results interpretation, the plasma biomarkers and MR imaging indices were Z‐scored. Z‐scores for each plasma biomarker and MRI variable were derived by calculating the mean (μ) and standard deviation (σ) separately for each biomarker and MRI variable in the full study sample using the formula z = (x – μ)/σ. This transformation standardized the distribution of each plasma biomarker and MRI variable such that the mean was zero and the standard deviation was 1. Secondary analyses examined (1) brain region‐specific associations (by lobe), (2) the modifying effect of age on the plasma biomarker‐MWF relationship using a continuous age*MRI‐biomarker interaction term, and (3) examined the effect of additional adjustment for cardiovascular risk factors (i.e., body mass index [BMI] and hypertension). All analyses were performed using MATLAB (MathWorks, Natick, MA, USA). To correct for Type I error, we used Bonferroni correction. Because each MRI variable was assessed in relation to four biomarkers (independent variables) we used a *P* < 0.013 (0.05/4 = 0.013) to denote statistical significance. A nominal *P* < 0.05 was considered a suggestive association.

## Results

### Cohort characteristics, plasma biomarkers and age

**Figure 2:**
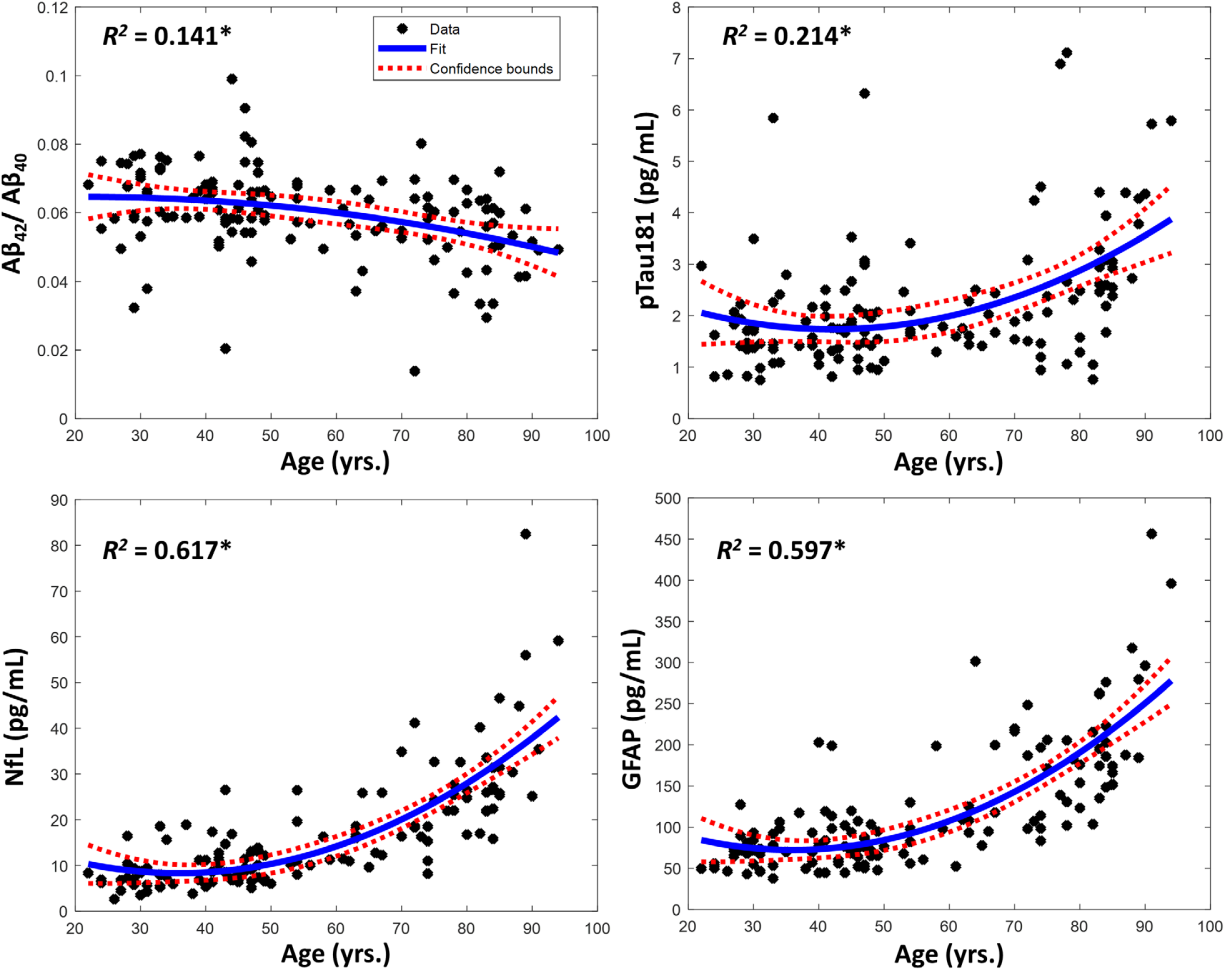
Association of Alzheimer's disease and neurodegeneration plasma biomarkers with age. Lines of best fit, confidence intervals and regression coefficients of determination are displayed. For each plot, line of fit and confidence intervals, as well as the regression coefficient of determination is displayed. Age‐biomarker associations were derived using a regression model incorporating a quadratic age term (i.e. polynomial regression of degree 2). *Indicates significance at *P* < 0.01. All associations remained statistically significant after Bonferroni correction for multiple comparisons.

A total of 119 participants (mean [SD] age, 55.2 [20.5] years; age range 22–94 years) were included in the current analysis of MRI‐defined MWF (Tables 1 and S1). A subset of these participants (*N* = 43) also underwent MRI for NDI measurement (mean [SD] age, 49.7 [18.3] years) and were included in all NDI analyses. Of the 119 participants included in the analytic sample, all were cognitively normal, i.e., without a diagnosis of mild cognitive impairment or dementia. As displayed in Figure 2, all neurodegenerative disease biomarkers demonstrated a significant nonlinear association with age. NFL and GFAP demonstrated especially strong age associations beginning around 50 years, whereas the Aβ_42/40_ and pTau181 age‐related associations remained relatively modest across the age span.

**Table 1:** Baseline participant characteristics.

Characteristic	NDI Sample (*N* = 43)	MWF Sample (*N* = 119)
Demographic Variables		
Age, years, mean (SD) [min–max]	49.7 (18.3) [24–83]	55.2 (20.5) [22–94]
Female, no (%)	22 (51.2)	54 (45.4)
White Race, no. (%)	30 (69.8)	82 (68.9)
Non‐White Race, no. (%)	13 (30.2)	37 (31.1)
Education, no. (%)		
High School/GED	7 (16.3)	19 (16.0)
Two Year College/Associates	4 (9.3)	11 (9.2)
Four Year College	18 (41.9)	38 (31.9)
Graduate Degree	12 (27.9)	44 (37.0)
Unknown/Other	2 (4.7)	7 (5.8)
BLSA Study, no. (%)	11 (25.6)	66 (55.5)
GESTALT Study, no. (%)	32 (74.4)	53 (44.5)
Clinical Variables, no. (%)		
Hypertension	4 (9.3)	26 (22.0)
Diabetes mellitus	0 (0.0)	3 (2.5)
BMI	25.6 (3.4)	25.9 (3.8)
MMSE, mean (SD)	29.2 (1.0)	28.8 (1.4)

Values are displayed as means (standard deviation) or frequencies (percentages). One participant is missing hypertension data; one participant missing diabetes data; two participants are missing BMI data; four participants are missing MMSE data. *Abbreviations*: MCI, mild cognitive impairment; MMSE, Mini‐Mental State Exam.

### Plasma neurodegenerative disease biomarkers and myelin water fraction

**Figure 3:**
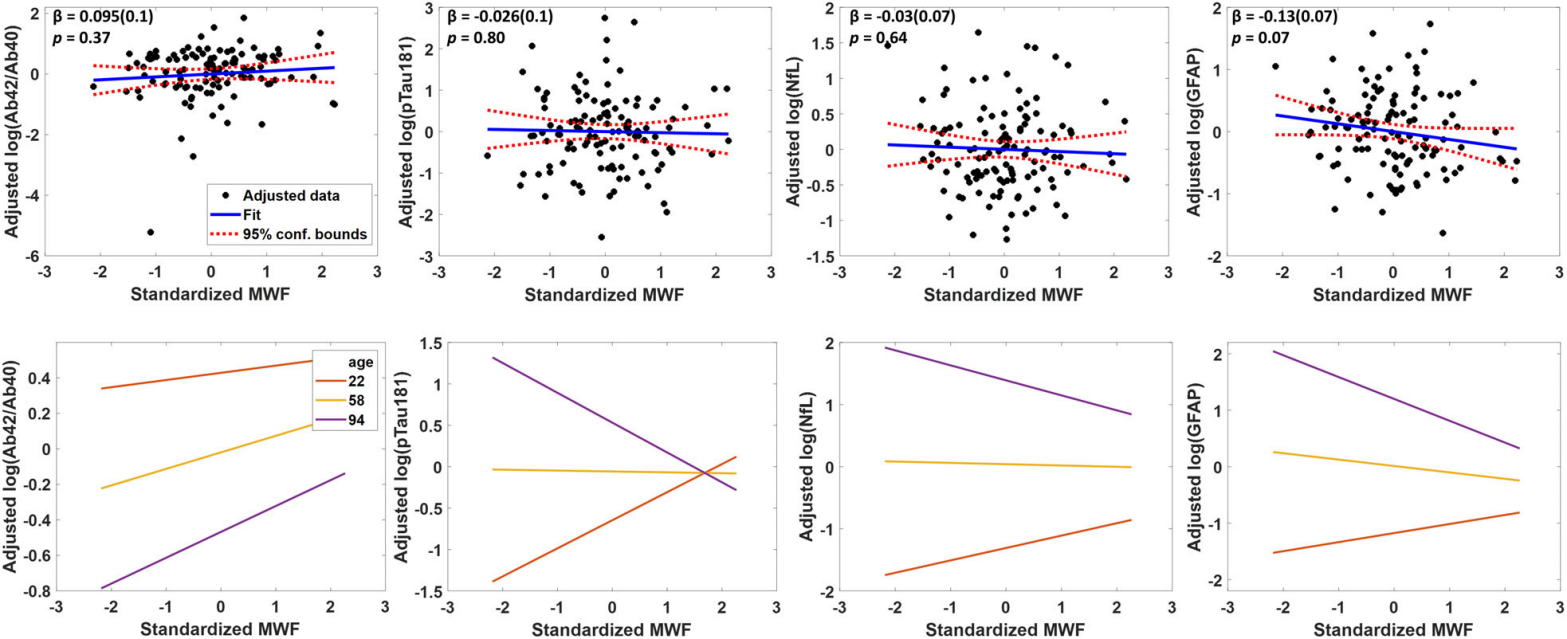
Association of Alzheimer's disease and neurodegeneration plasma biomarkers with whole brain myelin water fraction. The line of fit, regression coefficient (standard error), and *P*‐value were derived from the linear regression model adjusted for age, sex, and race (top row). The bottom row illustrates age‐stratified association between each plasma biomarker and brain myelin water fraction (MWF; bottom row) derived using a linear regression model adjusted for sex and race. The age‐stratified lines show the adjusted biomarker level as a function of MWF, with age fixed at three specific values of 22 (red line), 58 (yellow line), and 94 (purple line) corresponding to the minimal, median, and maximal values, respectively. All age‐stratified associations were nonsignificant. The adjusted response function describes the relationship between the fitted response and MWF, with the other predictors averaged out by averaging the fitted values over the data used in the fit.

After adjusting for age, sex and race, we found no relationship between plasma biomarkers and MWF derived from the whole brain (Fig. 3). However, lobar‐specific analyses showed a suggestive association between higher GFAP and lower MWF in the temporal lobe (*ß* = ‐0.13; SE = 0.06; *P =* 0.049) with similar but nonsignificant trends observed for the parietal and occipital lobes (Table S2). These associations were generally strengthened after adjustment for cardiovascular risk factors (Table S3). Notably, we found that age modified the association of pTau181 (interaction‐*P =* 0.05), NfL (interaction‐*P* = 0.06), and GFAP (interaction‐*P* = 0.02) with brain MWF. Exploratory analyses demonstrated that higher levels of pTau181, NfL, and GFAP tended to be associated with lower brain MWF among old, but not young or middle age, participants (Fig. 3).

### Plasma neurodegenerative disease biomarkers and axonal density

**Figure 4:**
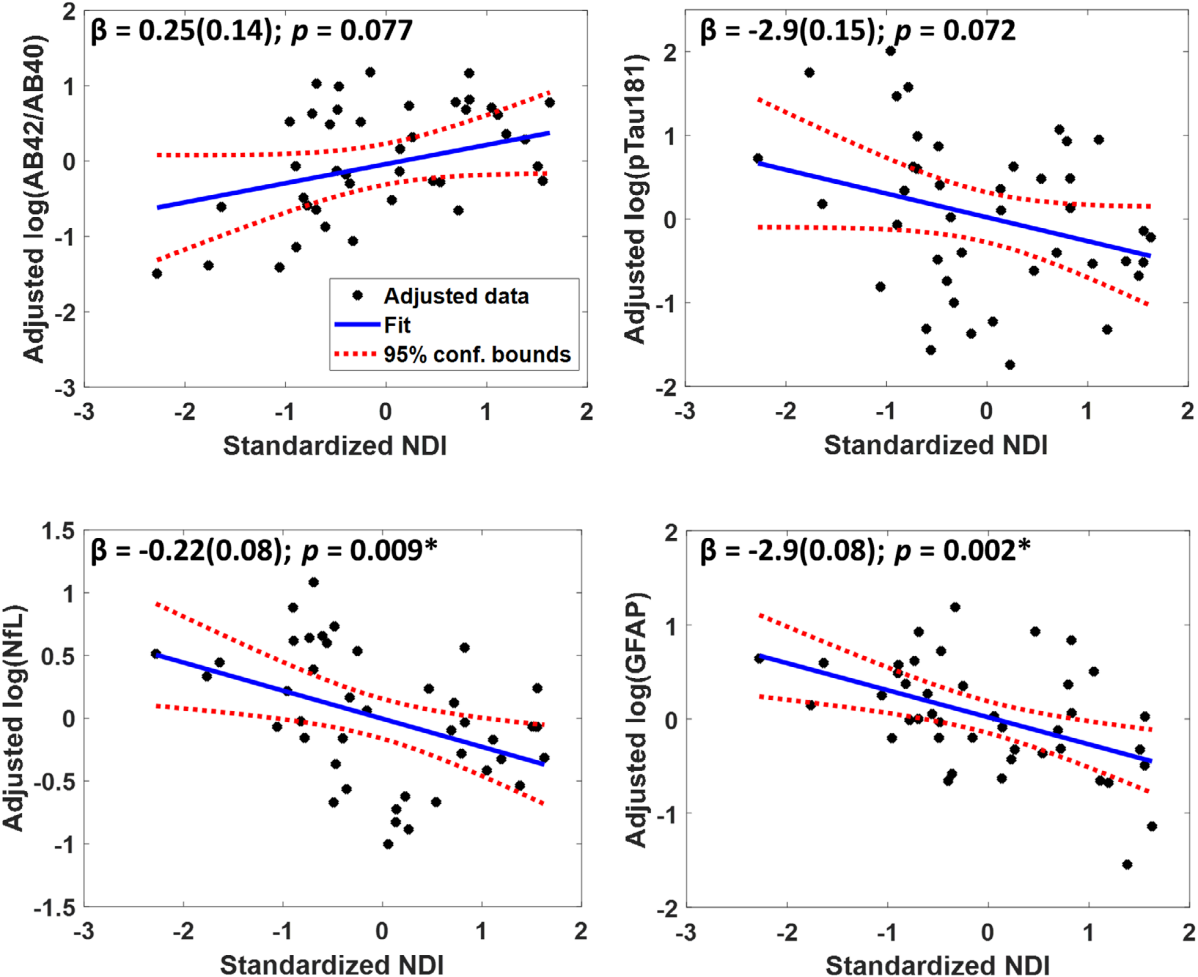
Association of Alzheimer's disease and neurodegeneration plasma biomarkers with whole brain axonal density (NDI). Line of fit and confidence intervals, as well as the regression coefficient, standard error, and *P*‐values were derived from the linear regression model adjusted for age, sex, and race (*N* = 43). *Indicates associations are statistically significant after Bonferroni correction for multiple comparisons (*P* < 0.013).

After adjusting for age, sex and race, elevated levels of NfL and GFAP were significantly associated with lower axonal density (NDI) derived from the whole brain white matter (Fig. 4). Though not statistically significant, we saw a trend toward associations of higher pTau181 and lower Aβ_42/40_ with lower axonal density/NDI as well; the magnitude of association with NDI was similar across the four AD/neurodegeneration biomarkers. The significant association between NfL and GFAP remained similar after additionally adjusting for cardiovascular risk factors (Table S4). Notably, with cardiovascular risk factor adjustment, the association of higher pTau181 and lower Aβ_42/40_ with lower axonal density/NDI was strengthened and demonstrated a suggestive association.

We next examined the lobar‐specific associations between plasma biomarkers and brain axonal density (Table 2). Elevated plasma NfL and GFAP were associated with lower axonal density in the frontal, temporal, and parietal lobes. Though not associated with total brain axonal density, lower plasma Aβ_42/40_ (indicative of more cortical amyloid) demonstrated a suggestive association with lower axonal density in the temporal and occipital brain regions, whereas elevated plasma pTau181 demonstrated a suggestive association with reduced axonal density in the occipital lobe. These results were similar after additionally adjusting for cardiovascular risk factors (Table S5). We did not examine age as an effect modifier given the modest sample size.

**Table 2:** Association of Alzheimer's disease and neurodegeneration plasma biomarkers with lobar axonal density (NDI).

Biomarker	Frontal Lobe NDI (*N* = 43)		Temporal Lobe NDI (*N* = 43)		Parietal Lobe NDI (*N* = 43)		Occipital Lobe NDI (*N* = 43)	
	*β* Estimate (SE)	*P*	*β* Estimate (SE)	*P*	*β* Estimate (SE)	*P*	*β* Estimate (SE)	*P*
Aβ_42/40_	0.23 (0.14)	0.113	0.29 (0.14)	0.043	0.23 (0.14)	0.121	0.35 (0.13)	0.014
pTau181	−0.21 (0.15)	0.175	−0.28 (0.15)	0.072	−0.22 (0.16)	0.174	−0.32 (0.15)	0.044
NfL	−0.23 (0.08)	0.007*	−0.22 (0.08)	0.012*	−0.25 (0.08)	0.004*	−0.19 (0.08)	0.026
GFAP	−0.31 (0.08)	<0.001*	−0.27 (0.09)	0.004*	−0.30 (0.09)	0.001*	−017 (0.09)	0.076

We used a linear regression model adjusted for age, sex and race for all analyses. β estimates represent the SD difference in plasma biomarker per each SD increase in lobar axonal density (NDI). *Abbreviations*: Aβ_42/40_, ratio of amyloid‐beta 42 to amyloid‐beta 40; GFAP, glial fibrillary acidic protein; NfL, neurofilament light chain; pTau181, tau phosphorylated at threonine‐181; SE, standard error.

* Indicates associations are statistically significant after Bonferroni correction for multiple comparisons (*P* < 0.013).

## Discussion

Aging and neurodegenerative disease lead to structural and functional changes to cerebral white matter that have downstream effects on cognitive and functional abilities. Though much research has focused on understanding the causes and consequences of diffusion‐based white matter microstructural changes, little is known about the specific roles of myelin content and axonal density in neurodegenerative diseases and their relationship to key biomarkers, particularly before the onset of cognitive impairment. Using plasma biomarkers and state‐of‐the‐art MR techniques, the current study demonstrates that elevated measures of neuronal injury (NfL) and reactive astrogliosis (GFAP) are associated with reduced total brain and lobar‐specific axonal density (NDI) in cognitively normal adults. Plasma Aβ_42/40_ and pTau181, measures of AD pathology, were also associated with lower axonal density, but only in select brain regions. In contrast, we found no significant relationship between neurodegenerative disease biomarkers and measures of myelin content (MWF), with the exception of an association between higher GFAP and reduced myelin content in the temporal lobe.

Although several studies have examined the association between plasma biomarkers of neurodegeneration and the integrity of cerebral white matter defined using MRI,^37^, ^38^, ^39^ these studies have not been conducted in the context of normative aging and have relied primarily on volumetry or diffusion tensor imaging indices. Although sensitive to myelin content and axonal density, these metrics are not specific. Therefore, to our knowledge, the specific associations of myelin content and axonal damage with plasma biomarkers of neurodegenerative processes have not been well characterized. Advanced MR methods based on multicomponent relaxometry or diffusion to assess MWF and NDI, as used here, have led to much greater specificity in noninvasive MR imaging of myelin content or axonal density. Measures of MWF (i.e., using BMC‐mcDESPOT), and NDI (i.e., using NODDI) have been shown to be very sensitive to capturing differences in myelin content and axonal density with aging, as well as their associations with various reversible or non‐reversible metabolic, genetic and vascular risk factors.^12^, ^29^, ^40^

The role of myelin and axonal loss in the context of aging and dementia has gained increasing attention in recent years.^41^, ^42^, ^43^ Myelin loss is hypothesized by some to be a key feature of AD and vascular cognitive impairment and dementia (VCID),^42^, ^43^, ^44^ and a recent study from our group found evidence of reduced myelin content among individuals with MCI and dementias.^14^ Supporting the link between myelin content and AD, a recent study found lower MWF‐defined myelin content accompanied increasing AD pathology, as defined by pTau181, pTau181/Aβ_42_, and several other biomarkers in CSF.^27^ The lack of association between MWF and biomarkers of AD pathology in the current study may be due to the inclusion of younger adults in our study sample, as we found that the direction of the MWF‐AD biomarker association changed with age.

A strength of the current study is the participant age range, which allowed us to determine age‐related variation in the plasma biomarker/myelin content relationship. NfL and GFAP demonstrated a nonlinear relationship with age with evidence for strong relationships between these biomarkers and age in participants 50 and older. Previous work from our group indicates that myelin content and axonal density (defined using MWF and NDI, respectively) begin to steadily decline around the same age range,^29^, ^45^ suggesting a similar temporal dynamic to that of the neuronal injury and astrogliosis markers observed in the current study. While there was no overall relationship between neurodegenerative disease biomarkers and myelin content after accounting for age and other demographic factors, we found that age appears to modify the relationship between myelin content and these biomarkers, particularly for pTau181, NfL, and GFAP. Therefore, studies with larger samples of older adults are warrented to more definitively determine whether AD pathology, neurodegeneration, and astrogliosis are associated with myelin loss in this age group.

Previous studies have shown that myelin abnormalities co‐localize with Aβ plaque deposition and correlate with plaque size in AD mouse models of Aβ. These findings support the direct association between Aβ and demyelination.^46^ Other studies have shown that iron deposition catalyzes Aβ and tau aggregations,^47^ while also being associated with myelin breakdown in the context of neurodegeneration and normative aging.^48^, ^49^ Therefore, the association between Aβ, tau, and myelin content was expected. However, further studies using PET imaging and CSF biomarkers, especially in the context of more advanced neurodegeneration, are required to further shed the light on these potential underlying mechanisms. The relationship between plasma GFAP and lower myelin content in the temporal lobe underscores the well documented relationship between astrocyte injury, demyelination, and oligodendrocyte degeneration observed in inflammatory conditions of the CNS, such as neuromyelitis optica.^50^

In contrast to MWF, NDI‐defined axonal density exhibited a robust inverse association with plasma biomarkers of neuronal injury and reactive astrogliosis – two cellular processes that occur across multiple neurodegenerative conditions. To the best of our knowledge, this is the first study to demonstrate a relationship between NDI‐defined axonal density and plasma NfL or GFAP among cognitively normal adults. By demonstrating this association between NDI and markers of neuronal injury and reactive astrogliosis, these findings support previous work which has suggested that axonal density declines in the context of other neurodegenerative processes, such as cortical tau deposition, neuroinflammation, and clinically defined AD.^30^, ^31^ Our finding that Aβ_42/40_ and pTau181 showed only lobar‐specific relationships with axonal density, particularly in temporal and occipital regions, suggests that axonal density may decline globally in relation to non‐specific processes such as neuronal injury or astrogliosis, yet decline regionally in relation to Alzheimer's neurodegenerative processes, particularly in cognitively unimpaired individuals.

An outstanding question that cannot be addressed using an observational study design is whether the relationships between greater neuronal injury/astrogliosis and reduced axonal density are mechanistic or merely associative. NfL, which is commonly classified as an indicator of neuronal injury, is a cytoskeleton scaffolding protein expressed primarily by large‐caliber myelinated axons.^51^ NfL may therefore simply be an indicator of axonal loss, and perhaps myelin loss, by virtue of its sensitivity to the various causes of neuronal injury, such as cerebral small vessel disease^52^ and neuroinflammation,^53^, ^54^ which are also suspected to promote axonal loss. While reactive astrogliosis is now known to occur early in the course of neurodegenerative disease,^55^ it remains unclear whether the reactive astrocytic phenotype(s) promote axonal loss. Although the mechanistic link remains unknown, the current study suggests that plasma GFAP may also serve as an indicator of axonal loss among cognitively normal adults.

The present cross‐study provides a foundation for future investigations, including longitudinal assessments, to clarify the extent to which plasma biomarkers of AD and neurodegenerative pathology reflect biologically meaningful changes in white matter structure and function defined using MRI. However, there are several study limitations that need to be considered. First, the study has a limited sample size, especially for the subsample of participants with NDI measures. This is a common feature of studies that employ novel or experimental neuroimaging sequences and has the effect of limiting our detection of statistical significance to only large effect sizes. Given the limited statistical power, null associations – particularly in subgroup analyses – should be interpreted with caution. Second, although this study was designed to examine plasma biomarkers, these biomarkers have important caveats that should be considered. Despite considerable improvements in plasma biomarker sensitivity with the advent of ultra‐sensitive immunoassays and advancement of mass spectrometry, plasma biomarkers – with the exception of plasma GFAP – remain less accurate than CSF biomarkers of the same molecule for discriminating individuals with AD pathological changes from those without.^55^, ^56^ Aß_42/40_ and pTau181 immunoassays show only moderate correlations with CSF measures of the same protein, though they have demonstrated fair to excellent classification of abnormal amyloid and tau, as measured by CSF, PET, or autopsy.^55^, ^56^, ^57^ Accordingly, the lack of an association between Aβ_42/40_ ratio, pTau181, NfL and myelin content may be due to measurement error and should be interpreted with caution. More generally, discrepancies between the current findings and results from previous studies that have relied on CSF biomarkers may be attributed to the differential sensitivity of CSF vs. plasma assays. Despite these limitations, the current study suggests that plasma biomarkers of neuronal injury (NfL) and reactive astrogliosis (GFAP) capture information about axonal density in cognitively normal adults. These results indicate further that reduced in axonal density may occur in conjunction with neuronal injury and astrogliosis, even before the emergence of clinically significant cognitive decline.

## Author Contributions

The following authors contributed to (1) The conception and design of the study: KAW, MB; (2) Acquisition and analysis of data: MRD, ZG, AL, ARM, SMR, MB (3) Drafting and revising the manuscript or figures: KAW, MRD, ZG, HED, JPL, MEF, YA, AL, ARM, SMR, MB.

## Funding Information

No funding information provided.

## Conflict of Interest

The authors report no conflicts of interest.

## Supporting information


**Table S1.** Baseline participant characteristics
**Table S2.** Association of Alzheimer's disease and neurodegeneration plasma biomarkers with lobar myelin water fraction (MWF).
**Table S3.** Association of Alzheimer's disease and neurodegeneration plasma biomarkers with lobar myelin water fraction (MWF) after adjusting for cardiovascular risk factors
**Table S4.** Association of Alzheimer's disease and neurodegeneration plasma biomarkers with whole brain axonal density (NDI).
**Table S5.** Association of Alzheimer's disease and neurodegeneration plasma biomarkers with lobar axonal density (NDI) after adjusting for cardiovascular risk factors.
