# Supplementary material for: MRI and fluid biomarkers reveal determinants of myelin and axonal loss with aging
Source: Ann Clin Transl Neurol. 2023 Feb 10;10(3):397–407. doi: 10.1002/acn3.51730 (PMC10014005; doi:10.1002/acn3.51730)
Supplement: Supplementary file 1 — Table S1. Baseline participant characteristics Table S2. Association of Alzheimer's disease and neurodegeneration plasma biomarkers with lobar myelin water fraction (MWF). Table S3. Association of Alzheimer's disease and neurodegeneration plasma biomarkers with lobar myelin water fraction (MWF) after adjusting for cardiovascular risk factors Table S4. Association of Alzheimer's disease and neurodegeneration plasma biomarkers with whole brain axonal density (NDI). Table S5. Association of Alzheimer's disease and neurodegeneration plasma biomarkers with lobar axonal density (NDI) after adjusting for cardiovascular risk factors. [file ACN3-10-397-s001.pdf]

Supplementary Materials

MRI and fluid biomarkers reveal determinants of myelin and axonal loss with aging

Keenan A. Walker, Michael R. Duggan, Zhaoyuan Gong, Heather E. Dark, John P. Laporte, Mary E. Faulkner, Yang An, Alexandria Lewis, Abhay R. Moghekar, Susan M. Resnick, Mustapha Bouhrara

Contents

Supplementary Table 1. Baseline participant characteristics 2

Supplementary Table 2. Association of Alzheimer's disease and neurodegeneration plasma biomarkers with lobar myelin water fraction (MWF) 3

Supplementary Table 3. Association of Alzheimer's disease and neurodegeneration plasma biomarkers with lobar myelin water fraction (MWF) after adjusting for cardiovascular risk factors 4

Supplementary Table 4. Association of Alzheimer's disease and neurodegeneration plasma biomarkers with whole brain axonal density (NDI). 5

Supplementary Table 5. Association of Alzheimer's disease and neurodegeneration plasma biomarkers with lobar axonal density (NDI) after adjusting for cardiovascular risk factors. 6

Supplementary Table 1. Baseline participant characteristics

Characteristic	BLSA (N = 66)	GESTALT (N = 53)
Demographic Variables		
Age, years, mean (SD)	54.7 (22.1)	55.8 (18.4)
Female, no (%)	33 (50.0)	21 (39.6)
White Race, no. (%)	41 (62.1)	41 (77.4)
Non-White Race, no. (%)	25 (37.9)	12 (22.6)
Education		
High School/GED	4 (6.1)	15 (28.3)
Two Year College/Associates	6 (9.1)	5 (9.4)
Four Year College	22 (33.3)	16 (30.2)
Graduate Degree	29 (43.9)	15 (28.3)
Unknown/Other	5 (7.5)	2 (3.8)
MWF Study, no. (%)	66 (100.0)	53 (100.0)
NDI Study, no. (%)	11 (16.7)	32 (60.4)
Clinical Variables, no. (%)		
Hypertension	20 (30.3)	6 (11.3)
Diabetes mellitus	3 (4.5)	0 (0.0)
BMI	25.9 (4.1)	25.8 (3.3)
MMSE, mean (SD)	28.6 (1.5)	29.1 (1.2)

Values are displayed as means (standard deviation) and frequencies (percentages). One participant is missing hypertension data; one participant missing diabetes data; two participants are missing BMI data; four participants are missing MMSE data.
Abbreviations: MCI, mild cognitive impairment; MMSE, Mini-Mental State Exam

Supplementary Table 2. Association of Alzheimer's disease and neurodegeneration plasma biomarkers with lobar myelin water fraction (MWF).

Biomarker	Frontal Lobe MWF (N = 119)		Temporal Lobe MWF (N = 119)		Parietal Lobe MWF (N = 119)		Occipital Lobe MWF (N = 119)	
	β Estimate (SE)	*P*	β Estimate (SE)	*P*	β Estimate (SE)	*P*	β Estimate (SE)	*P*
$A\beta_{42/40}$	0.06 (0.11)	0.56	0.10 (0.10)	0.32	0.18 (0.10)	0.09	0.17 (0.09)	0.08
pTau181	-0.06 (0.10)	0.56	-0.01 (0.09)	0.91	-0.07 (0.10)	0.51	0.01 (0.09)	0.91
NfL	-0.02 (0.07)	0.74	-0.02 (0.06)	0.68	-0.05 (0.06)	0.42	-0.05 (0.05)	0.37
GFAP	-0.12 (0.07)	0.10	-0.13 (0.06)	0.049	-0.13 (0.07)	0.06	-0.12 (0.06)	0.06

We used a linear regression model adjusted for age, sex, and race for all analyses. β estimates represent the SD difference in plasma biomarker per each SD increase in lobar myelin water fraction (MWF).

No results were statistically significant after Bonferroni correction for multiple comparisons ($P<0.013$).

Abbreviations: $A\beta_{42/40}$, ratio of amyloid-beta 42 to amyloid-beta 40; GFAP, glial fibrillary acidic protein; NfL, neurofilament light chain; pTau181, tau phosphorylated at threonine-181; SE, standard error.

Supplementary Table 3. Association of Alzheimer's disease and neurodegeneration plasma biomarkers with lobar myelin water fraction (MWF) after adjusting for cardiovascular risk factors

Biomarker	Total Brain MWF (N = 119)		Frontal Lobe MWF (N = 119)		Temporal Lobe MWF (N = 119)		Parietal Lobe MWF (N = 119)		Occipital Lobe MWF (N = 119)	
	β Estimate (SE)	*P*	β Estimate (SE)	*P*	β Estimate (SE)	*P*	β Estimate (SE)	*P*	β Estimate (SE)	*P*
$A\beta_{42/40}$	0.11 (0.11)	0.32	0.08 (0.11)	0.47	0.10 (0.10)	0.30	0.20 (0.10)	0.06	0.18 (0.09)	0.06
pTau181	-0.04 (0.10)	0.70	-0.08 (0.11)	0.47	-0.02 (0.1)	0.82	-0.08 (0.10)	0.42	-0.02 (0.09)	0.83
NfL	-0.07 (0.06)	0.28	-0.07 (0.07)	0.28	-0.06 (0.06)	0.32	-0.10 (0.06)	0.14	-0.08 (0.05)	0.17
GFAP	-0.14 (0.07)	0.06	-0.14 (0.07)	0.08	-0.14 (0.06)	0.04	-0.15 (0.07)	0.04	-0.13 (0.06)	0.05

We used a linear regression model adjusted for age, sex, race, hypertension (yes/no), and BMI (defined continuously).
β estimates represent the SD difference in plasma biomarker per each SD increase in lobar myelin water fraction (MWF).
No results were statistically significant after Bonferroni correction for multiple comparisons (P<0.013).
Abbreviations: $A\beta_{42/40}$, ratio of amyloid-beta 42 to amyloid-beta 40; GFAP, glial fibrillary acidic protein; NfL, neurofilament light chain; pTau181, tau phosphorylated at threonine-181; SE, standard error.

Supplementary Table 4. Association of Alzheimer's disease and neurodegeneration plasma biomarkers with whole brain axonal density (NDI).

Biomarker	Primary Model (N = 43)		Risk Factor Model (N = 43)	
	β Estimate (SE)	*P*	β Estimate (SE)	*P*
$A\beta_{42/40}$	0.25 (0.14)	0.08	0.34 (0.16)	0.04
pTau181	-0.29 (0.15)	0.07	-0.42 (0.15)	0.02
NfL	-0.22 (0.08)	0.009*	-0.21 (0.09)	0.03
GFAP	-0.29 (0.08)	0.002*	-0.29 (0.10)	0.008*

Results were derived using a linear regression model. The primary model adjusted for age, sex, and race. The risk factor model adjusted for age, sex, race, hypertension (yes/no), and BMI (defined continuously). β estimates represent the SD difference in plasma biomarker per each SD increase in lobar axonal density (NDI).

* Indicates associations are statistically significant after Bonferroni correction for multiple comparisons ($P<0.013$).

Abbreviations: $A\beta_{42/40}$, ratio of amyloid-beta 42 to amyloid-beta 40; GFAP, glial fibrillary acidic protein; NfL, neurofilament light chain; pTau181, tau phosphorylated at threonine-181; SE, standard error.

Supplementary Table 5. Association of Alzheimer's disease and neurodegeneration plasma biomarkers with lobar axonal density (NDI) after adjusting for cardiovascular risk factors.

Biomarker	Frontal Lobe NDI (N = 43)		Temporal Lobe NDI (N = 43)		Parietal Lobe NDI (N = 43)		Occipital Lobe NDI (N = 43)	
	β Estimate (SE)	*P*	β Estimate (SE)	*P*	β Estimate (SE)	*P*	β Estimate (SE)	*P*
$A\beta_{42/40}$	0.30 (0.16)	0.08	0.35 (0.15)	0.03	0.31 (0.16)	0.07	0.37 (0.14)	0.02
pTau181	-0.29 (0.17)	0.10	-0.36 (0.16)	0.03	-0.36 (0.17)	0.04	-0.33 (0.16)	0.05
NfL	-0.22 (0.09)	0.02	-0.20 (0.09)	0.03	-0.27 (0.09)	0.004*	-0.17 (0.09)	0.06
GFAP	-0.33 (0.10)	0.002*	-0.25 (0.10)	0.02	-0.31 (0.10)	0.003*	-015 (0.10)	0.13

We used a linear regression model adjusted for age, sex, race, hypertension (yes/no), and BMI (defined continuously) for all analyses.
β estimates represent the SD difference in plasma biomarker per each SD increase in lobar axonal density (NDI).
* Indicates associations are statistically significant after Bonferroni correction for multiple comparisons (P<0.013).
Abbreviations: $A\beta_{42/40}$, ratio of amyloid-beta 42 to amyloid-beta 40; GFAP, glial fibrillary acidic protein; NfL, neurofilament light chain; pTau181, tau phosphorylated at threonine-181; SE, standard error.
